# The Complex Consequences of Severe Polytrauma with Traumatic Brain Injuries Caused by a Traffic Accident in a Child: A Case Report

**DOI:** 10.3390/children12040516

**Published:** 2025-04-17

**Authors:** Liliana Anghelina, Lucrețiu Radu, Cristian Gheonea, Vlăduț Teică, Adelina-Maria Anghelina, Mirela Anişoara Siminel

**Affiliations:** 1 Department of Pediatrics, University of Medicine and Pharmacy of Craiova, 200638 Dolj, Romania; 2 Department of Hygiene, University of Medicine and Pharmacy of Craiova, 200638 Dolj, Romania; 3 Department of Biophysics, University of Medicine and Pharmacy of Craiova, 200638 Dolj, Romania; 4 Department of Radiology and Medical Imaging, University of Medicine and Pharmacy of Craiova, 200638 Dolj, Romania; 5 Medical School, University of Medicine and Pharmacy of Craiova, 200638 Dolj, Romania; 6 Department of Neonatology, University of Medicine and Pharmacy of Craiova, 200638 Dolj, Romania

**Keywords:** polytrauma, cranio-cerebral trauma, syncope, psychosocial disabilities, case report

## Abstract

Background. Polytrauma is one of the three leading causes of mortality worldwide and a major contributor to psychosocial morbidity and disability. Concomitant traumatic brain injuries further increase this risk. Methods. We present the case of a 6-year-old child who sustained significant trauma in a road accident, resulting in injuries to multiple anatomical regions, including the central nervous system. Results. Two years after the accident, the child experiences brief episodes of decreased visual acuity, dizziness, nausea, pallor, and headaches, along with occasional migraines that resolve spontaneously. Additionally, the child struggles with school adjustment. Brain injuries associated with polytrauma are crucial prognostic factors in both the short and long term, influenced by the brain’s reactivity and recovery capacity. There is also an increased risk of developing syncopal episodes, seizures, and other neurological manifestations. Conclusions. The direct and secondary effects of the trauma have contributed to psychosocial disabilities, necessitating complex medical care and family-centered interventions.

## 1. Introduction

Polytrauma is a syndrome, a severe acute condition resulting from multiple injuries to tissues, organs, and systems, along with systemic functional impairments. Butcher and colleagues define polytrauma using an Abbreviated Injury Scale (AIS) score of ≥3 for at least two different body regions, with at least one injury being life-threatening and requiring complex, coordinated medical care [1]. The new Berlin definition of polytrauma also uses an AIS score of ≥3 for two or more different body regions, along with one or more additional variables from five physiological parameters: hypotension (≤90 mmHg), Glasgow Coma Scale score (≤8), base excess ≤ 6.0, international normalized ratio ≥ 1.4 or partial thromboplastin time ≥ 40 s, and age ≥ 70 years [2].

In children with polytrauma, multiorgan failure occurs early, with simultaneous failure of systems and organs, in contrast to the sequential and delayed onset of multiorgan failure in adults [3]. Trauma scores for adults with polytrauma are not universally applicable to children, making the prognostic assessment of pediatric patients with polytrauma more difficult [4]. The Pediatric Trauma Score (PTS) includes skeletal, cutaneous, and central nervous system injuries, as well as physiological parameters such as body weight, systolic blood pressure, and airway status. The value of PTS as a prognostic factor in children remains controversial [5].

The assessment of a child’s state of consciousness is based on scales derived from the Glasgow Coma Scale, adapted according to the degree of language development. These scales include the Pediatric Glasgow Coma Scale (PGCS), the Children Coma Scale (CCS), and the Infant and Toddler Coma Scale [6].

The magnitude of the impact energy, as well as the vulnerability of the host, determines the nature and severity of the injuries [7]. Children with severe forms of traffic accident polytrauma have the highest mortality rates and are at greater risk of developing disabilities [8]. Associated cranio-cerebral trauma is more frequent and severe and remains a significant issue [9,10].

In the context of imaging investigations for polytrauma, the first step is to perform a standard radiological examination of the skull, spine, and any other affected regions [11]. Cranio-cerebral computed tomography is considered the gold standard for investigating traumatic brain injuries. It is readily available in emergency situations and provides high-resolution visualization of hemorrhagic injuries, skull fractures, and dural or brain herniations through the bone [11]. CT scans are the best choice for imaging solid organs (such as the liver and spleen) and remain the gold standard for evaluating abdominal trauma in hemodynamically stable patients [12,13].

In the case of orthopedic injuries, the primary objective is to restore functionality, allowing the patient to return to daily activities. For polytrauma, functional recovery is more complex, requiring a comprehensive understanding of the entire spectrum of trauma care both during hospitalization and afterward. This includes considering the patient’s perception, age, and the family’s perspective on the trauma.

To improve the quality of care, it is essential to identify the factors that negatively affect the management of trauma in children and seek appropriate solutions. Polytrauma, which involves the full spectrum of injuries, is a major cause of severe illness due to its complexity and requires advanced care methods to reduce morbidity rates.

Post-traumatic symptoms may include loss of consciousness with a syncopal episode, seizures, severe headache accompanied by neurological manifestations, confusional syndrome, visual disturbances, and amnesic episodes.

In cases of cranio-cerebral trauma, three pathophysiological groups are identified:

1. Loss of consciousness with a syncopal episode: The clinical picture is similar to that of classic vagal syncope, where the child experiences a brief period of unconsciousness accompanied by intense pallor [14]. Vasovagal syncope can sometimes occur in adolescents. Pathophysiologically, it correlates with both vasovagal syncope and neurocardiogenic syncope [14]. In rare cases, loss of consciousness is followed by gradual recovery with regenerative sleep, clonic activity, classic vasovagal syncope complicated by short-term cerebral anoxia, and seizures [15,16,17].
2. Seizures, which can complicate 2% of cases of traumatic brain injury in children, usually occur within the first two hours after the injury and last less than five minutes. Occasionally, there is a personal history of febrile seizures, but the prognosis is favorable, and the cranial CT is normal [18,19,20]. In some cases, convulsions may be the first manifestation of epilepsy, with cranial trauma acting merely as a contributing factor or even a coincidence. As with all cases of non-lesional occasional convulsions (e.g., febrile seizures), the pathophysiological mechanism involves abrupt physicochemical changes in neuronal membranes and ion channels [21].
3. Prolonged neurological manifestations: The most common symptoms include headache and qualitative or quantitative alterations in consciousness. Other neurological changes include visual disturbances, amnesia, paresis, dysphasia, vertigo, dysautonomic signs (nausea, vomiting, and pallor), and secondary seizures [22,23]. Migraine can occur in children or teenagers. Complicated migraines may be associated with traumatic triggers, such as hemiplegic migraine, a condition referred to as ‘childhood post-traumatic migraine’ [19,22,23].

Physiologically, a migraine crisis involves a ‘threshold effect’, which refers to the varying degree of triggers. These may include common triggers or attacks that occur only under special conditions, such as the combination of young age and head trauma, which activates an unstable or immature trigeminovascular system [20,24].

Cranio-cerebral traumas often lead to cognitive disorders, manifesting as a decline in thinking ability and memory compared to previous functioning levels. The most common psychiatric complications include depression, generalized anxiety, and panic attacks [20,24].

The economic and social impact of children with polytrauma is significantly influenced, both directly and indirectly, by the costs of acute injury treatment, rehabilitation, and the long-term sequelae they experience [24,25,26]. Assessment and follow-up involve complex care and family-centered interventions to optimize long-term outcomes [27].

Psychological disorders that develop later in childhood following cranio-cerebral trauma are rarely diagnosed. Organic post-traumatic brain syndrome can lead to the deterioration of cognitive or executive functions despite a normal IQ, along with maladaptive behavioral disorders. As a result, periodic re-examinations are necessary for childhood cranial trauma, with expanded psychological evaluations and recommendations for rehabilitative treatment [28,29].

Polytraumatized patients present a challenge, and optimizing treatment methods and ensuring their correct management is essential [30].

This article highlights that polytrauma and brain injuries resulting from a childhood road traffic accident involve both immediate direct effects and complex long-term consequences. Over time, these consequences can lead to neurological manifestations and difficulties in adapting to school. Due to the interaction of factors related to the injury, environment, and family, it is essential to provide comprehensive care for the child within their family and social context to optimize treatment methods and case management.

## 2. Case Description

We present the case of a 6-year-old girl who was involved in a road accident and sustained multiple injuries to various anatomical regions, including trauma to the central nervous system, with a Glasgow Coma Scale score of 8, orotracheal intubation, and mechanical ventilation. As a result of the impact, her mother died at the scene of the accident.

**Figure 1:**
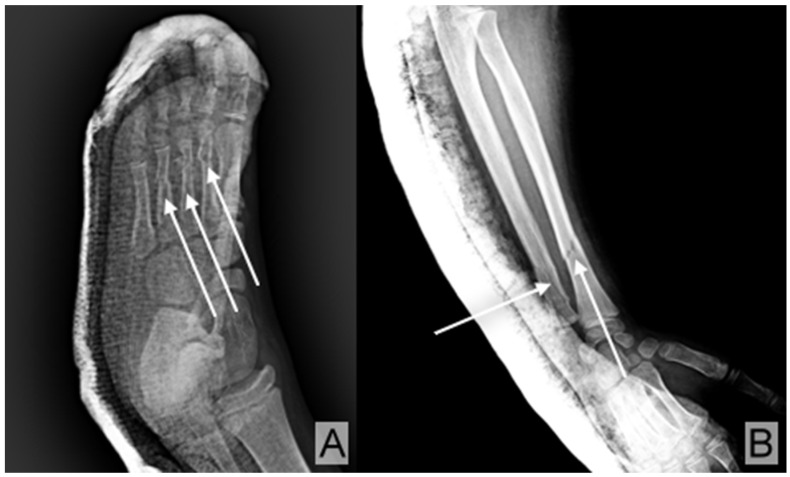
(**A**) X-ray of the right leg showing open fractures (arrows) of the second, third, and fourth metatarsals. (**B**) X-ray of the right forearm revealing fractures (arrows) of both the radius and ulna.

**Figure 2:**
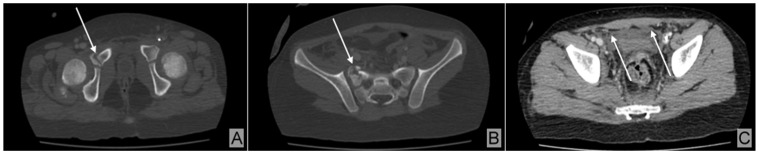
Pelvic CT scan findings. (**A**) Fractures of the pubic ramus and right sacral wing (arrow). (**B**) Displaced fracture of the right sacral wing (S1) (arrow), along with a fracture and displacement of the outer third of the right ilio-pubic ramus. (**C**) Hyperintense signal in the pelvic region adjacent to the fracture, indicating pelvic fat infiltration (arrows) due to the impact.

As part of the severe polytrauma, she presented with the following injuries: open fractures of the second, third, and fourth metatarsals of the right foot (Figure 1A); a fracture of both bones in the right forearm (Figure 1B); a contusion of the left elbow; fractures of the pubic ramus and right sacral wing (Figure 2A); a fracture with displacement of the right sacral wing (S1), along with a fracture and displacement of the outer third of the right ilio-pubic ramus (Figure 2B); and pelvic fat infiltration adjacent to the pelvic fracture, resulting from the impact (Figure 2C).

**Figure 3:**
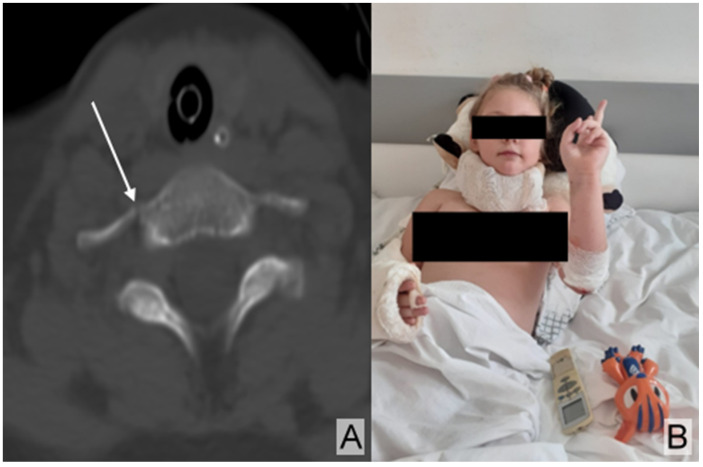
(**A**) Spine CT showing a fracture of the transverse process (arrow) of the seventh cervical vertebra with minimal displacement and no other detectable fracture lines in the cervical spine. (**B**) Patient immobilized in a cervical collar.

She also sustained a fracture of the seventh transverse process with minimal displacement (Figure 3A), which was immobilized in a cervical collar (Figure 3B).

**Figure 4:**
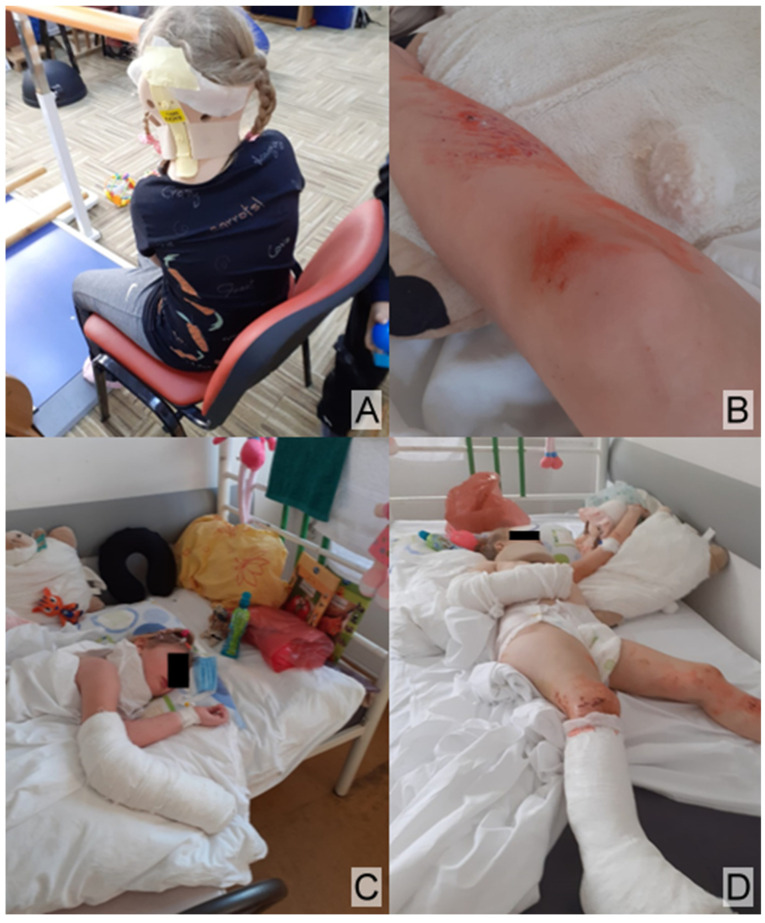
(**A**) Open wound on the scalp. (**B**) Multiple excoriations on the lower limbs. (**C**) Cast immobilization of the right brachiopalmar region. (**D**) Cast immobilization of the right foot.

Other injuries included multiple rib fractures, an open wound on the ankle communicating with a fracture, an open wound on the right elbow, multiple open wounds on the lower limbs, an open wound on the scalp (Figure 4A), and multiple excoriations (Figure 4B). A right brachiopalmar cast (Figure 4C) and a right foot cast for immobilization (Figure 4D) were applied.

**Figure 5:**
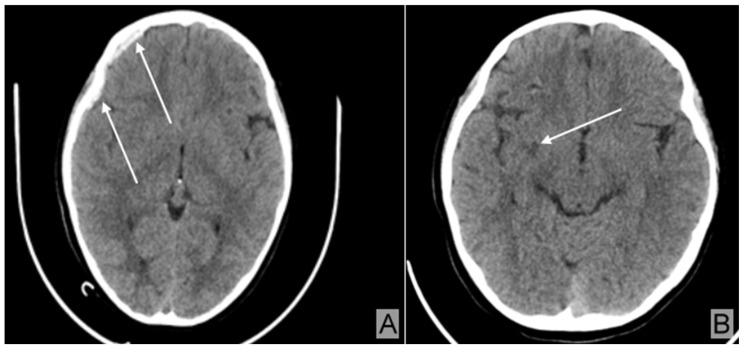
Skull CT: (**A**) A spontaneously hyperdense area measuring 60 mm in length and 4 mm in maximum thickness (arrows), exhibiting the characteristics of an acute subdural hematoma. (**B**) A spontaneously hypodense area measuring 4.8 mm in size (arrow) in the white matter of the right frontal region, suggestive of diffuse axonal injury.

She also sustained an acute subdural hematoma (Figure 5A) and a diffuse axonal injury (Figure 5B).

No focal lesions or abdominal fluid were detected on the abdominal CT. No focal lesions of the internal genital organs were detected, and there were no pelvic adenopathies. Pelvic peritoneal fluid was present on the pelvic CT. The lung parenchyma was normal, with no signs of pulmonary consolidation or suspicious nodules on the thoracic CT.

Initial management focused on saving the patient’s life by stabilizing the airway, breathing, and circulation, and providing care for the polytrauma in accordance with pediatric advanced life support (PALS) and advanced trauma life support (ATLS) protocols. To salvage limbs with severe open fractures, all wounds were covered with a dressing moistened with povidone–iodine solution (Betadine). The deformed limbs were aligned, fractures were temporarily stabilized with splints during initial resuscitation, and then the patient was transferred to the imaging room and intensive care unit, minimizing pain and early mobilization. In the presence of associated head trauma, hyperventilation of the intubated patient and fluid restriction were implemented. Tetanus prophylaxis was administered. Prophylactic intravenous antibiotics (cephalosporin and aminoglycoside) were given for 48 to 72 h, with an additional 48 to 72 h administered around subsequent surgeries. Intramedullary fixation with flexible titanium rods for open fractures of the long bones was performed within the first 6 to 8 h of injury. Once the child was stable enough to undergo surgery, closed fractures of the long bones were surgically stabilized between the 2nd and 3rd day.

Two years after the accident, the child experienced episodes of decreased visual acuity, dizziness, nausea, and headaches lasting 10–15 min, which remitted spontaneously, as well as rare migraine episodes.

Upon admission to our Pediatric Clinic, two years after the polytrauma, the general clinical examination revealed the following: height of 140 cm, weight of 55 kg, and a BMI (body mass index) of 28.2, indicating excess adipose tissue. The child also had numerous post-traumatic keloid scars on the lower limbs, as well as on the right elbow, right forearm, and occipital region. A clinical examination of the organs and systems revealed no pathological changes, and the blood pressure was age-appropriate.

Complete blood count, coagulation parameters, inflammatory markers, liver function tests, kidney function tests, serum ionogram, additional tests (PRL (Prolactin), FT4 (Free Thyroxine), TSH (Thyroid Stimulating Hormone), Anti-TPO (Thyroid Antibodies), Free Cortisol, ACTH (Adrenocorticotropic Hormone), and Hb A1c (Glycosylated Hemoglobin) exhibited normal values, while lipids (see Table 1) showed upregulated values.

Complex investigations, including tomography, were performed alongside consultations in ophthalmology, cardiology, neurology, and psychiatry.

Radiograph of the wrist showed that the bone age was consistent with the chronological age. The skull CT showed no evolving or secondary focal lesions. The magnetic resonance imaging (MRI) examination could not be performed as the child was unable to cooperate at the time.

The ophthalmological consultation did not reveal any pathological changes. The neurological examination showed normal findings. The awake electroencephalogram (EEG) was normal for the patient’s age. The pediatric cardiology consultation and echocardiography did not reveal any pathological abnormalities.

The Pediatric Psychiatry consultation showed that the child had reactive emotional disorders. She was conscious, oriented to time, and exhibited clear behavior. Her facial expressions and gestures were consistent with her emotional experiences. Her memory was normal, although she had painful memories related to the traumatic event. She reported missing her mother, who died in the accident. She also had difficulty adapting to school, stating that she had only one friend, a single classmate, and exhibited low self-esteem along with a need for appreciation and validation. After the accident and up until the time of presentation at our clinic, the child was only occasionally evaluated psychologically.

## 3. Discussion

In the presented case, at the age of six, the child suffered a severe acute condition resulting from a road accident that caused multiple injuries to various anatomical regions, including the central nervous system.

Imaging studies were performed as part of the critical condition assessment at the time of the accident to ensure the appropriate and timely care of seriously injured children [11].

The radiographs of the injured areas revealed various types of fractures (Figure 1A,B), some of which were displaced and associated with pelvic fat infiltration resulting from the impact (Figure 2A–C). These fractures required specialized treatment, including cast immobilization (Figure 4C,D) and dynamic monitoring of the healing process.

The CT scan of the cervical spine revealed a fracture of the transverse process of the seventh cervical vertebra, with minimal displacement and no other detectable fracture lines in the cervical spine (Figure 3A).

The skull CT examination revealed a spontaneously hypodense area of 4.8 mm in the right frontal white matter, most likely representing a diffuse axonal injury (Figure 5B), which increased the severity of the condition [9].

CT scan of the abdomen, pelvis, and thoracic region revealed no lesions in the abdominal organs or lung parenchyma. At the pelvic level, no lesions in the pelvic organs were observed; however, pelvic peritoneal fluid was observed, along with a displaced fracture of the right S1 sacral wing and a displaced fracture of the outer third of the right ilio-pubic ramus. In addition, an area in the pelvic region, adjacent to the fracture, showed pelvic fat infiltration resulting from the impact (Figure 2A–C).

Care in the presented case remains complex even after overcoming the critical phase, requiring a thorough understanding of the entire spectrum of trauma care, including its perception in relation to the patient’s age and the family’s understanding of the trauma [31].

After the accident, the severe injuries associated with this polytrauma resulted in multiple keloid scars. The fractures healed without functional or neurological impairment, and the child achieved motor and physical skills within the expected range for her age. She presented with mild weight gain and mild dyslipidemia, but no associated endocrine disorders were found. The wrist radiograph showed that the bone age was consistent with the chronological age. The skull CT scan showed no evolving or secondary focal lesions.

Post-traumatic symptomatology included syncopal episodes [14], as well as migraines, which presented as a moderate headache accompanied by other neurological symptoms such as visual disturbances and dysautonomic signs (nausea, vomiting, and pallor) [22,23]. The neurological examination revealed no abnormalities.

Cranio-cerebral trauma often leads to cognitive disorders, with the child experiencing difficulty adapting to school and exhibiting low self-esteem. This may require psychological counseling and psychiatric supervision [28,29].

To optimize long-term outcomes, it is crucial to assess and monitor the child within her family and social context. She is currently in the care of her father and paternal grandmother, following her mother’s death in this road accident. Therefore, periodic re-examinations, including comprehensive psychological and psychiatric assessments, as well as the initiation of rehabilitation treatment, are essential.

## 4. Conclusions

Polytrauma with associated head injury, even when successfully treated, remains a risk factor for subsequent symptoms. Cognitive, affective, and behavioral disorders can have a significant impact on the child, family, and society, leading to social dependency even in the absence of physical disabilities. These disorders affect school performance, family relationships, and daily activities. Therefore, neuropsychological recovery is crucial, as traumatic brain injury can signal the onset of a lifelong condition.

## Figures and Tables

**Table 1:** Tests with abnormal values.

Class/Parameter	Value
Triglycerides	113 mg/dL
HDL Cholesterol	34 mg/dL
LDL Cholesterol	122.4 mg/dL

## Data Availability

The data presented in this study are available on reasonable request from the corresponding author.
